# Interrelationships between Atopic Disorders in Children: A Meta-Analysis Based on ISAAC Questionnaires

**DOI:** 10.1371/journal.pone.0131869

**Published:** 2015-07-02

**Authors:** David H. J. Pols, Jorien B. Wartna, Elvira I. van Alphen, Heleen Moed, Nadine Rasenberg, Patrick J. E. Bindels, Arthur M. Bohnen

**Affiliations:** Department of General Practice, Erasmus MC, University Medical Center Rotterdam, Rotterdam, The Netherlands; Cincinnati Children's Hospital Medical center, UNITED STATES

## Abstract

**Purpose:**

To study the prevalence and interrelationship between asthma, allergic rhinitis and eczema using data obtained from ISAAC questionnaires.

**Method:**

The Medline, Pubmed Publisher, EMBASE, Google Scholar and the Cochrane Controlled Clinical Trials Register databases were systematically reviewed to evaluate epidemiological data of children with atopic disorders. To study these interrelationships, a new approach was used. Risk ratios were calculated, describing the risk of having two different atopic disorders when the child is known with one disorder.

**Results:**

Included were 31 studies, covering a large number of surveyed children (n=1,430,329) in 102 countries. The calculated worldwide prevalence for asthma, eczema and allergic rhinitis is 12.00% (95% CI: 11.99-12.00), 7.88% (95% CI: 7.88-7.89) and 12.66% (95% CI: 12.65-12.67), respectively. The observed prevalence [1.17% (95% CI: 1.17-1.17)] of having all three diseases is 9.8 times higher than could be expected by chance. For children with asthma the calculated risk ratio of having the other two disorders is 5.41 (95% CI: 4.76-6.16), for children with eczema 4.24 (95% CI: 3.75-4.79), and for children with allergic rhinitis 6.20 (95% CI: 5.30-7.27). No studied confounders had a significant influence on these risk ratios.

**Conclusions:**

Only a minority of children suffers from all three atopic disorders, however this co-occurrence is significantly higher than could be expected by chance and supports a close relationship of these disorders in children. The data of this meta-analysis supports the hypothesis that there could be a fourth distinct group of children with all three disorders. Researchers and clinicians might need to consider these children as a separate group with distinct characteristics regarding severity, causes, treatment or prognosis.

## Background

Eczema, asthma and allergic rhinitis are common atopic disorders among children, making it an important public health problem worldwide. The prevalences of these three disorders show variability at regional and even at country level [1–4]. Despite this variability, there seems to be a close relationship between these disorders. In a triad of events that include eczema, asthma and allergic rhinitis, eczema is often the first disorder to evolve. A biologically plausible pathway to explain this cascade was proposed by Burgess et al [5]. As a result of a defective skin barrier in children with eczema, an epicutaneous sensitization to an allergen can take place resulting in T-helper type 2 memory cells; these cells can migrate to nasal and bronchial lymphoid tissue. When the airways become exposed to the same allergen, this might cause asthma and/or allergic rhinitis symptoms to evolve. However, in practice, the number of patients following this classic ‘atopic march’ seems to vary considerably [6, 7], only partially explaining the interrelationships.

The International Study of Asthma and Allergies in Childhood (ISAAC) was established in 1991 and formally closed in December 2012. The ISAAC study was divided into three phases. The purpose was to assess the worldwide prevalence of asthma, allergic rhinitis and eczema in children in the open population and to obtain possible risk factors that could influence these three disorders using a standardized questionnaire [8–10]. This makes ISAAC a reliable data source to use when studying the interrelationship of atopic diseases in children aged 6–7 and 13–14 years. Although non-ISAAC research groups (i.e. non-official ISAAC studies) also published data using ISAAC questionnaires, the official ISAAC reviews do not include these latter data in their analyses.

The primary aim of this review is to calculate the worldwide prevalence in children of asthma, allergic rhinitis, eczema, and of having all three disorders, using data obtained with ISAAC questionnaires and to examine interrelationships between these disorders using risk ratios. Risk ratios will describe the risk of having two different atopic disorders when the child is known with one disorder. A secondary aim is to analyze whether these risk ratios and prevalences are influenced by potential confounders such as study period, age, sex, continent, and use of the original English-language ISAAC questionnaire.

## Methods

The protocol of this review is described below.

### Search strategy

An extensive literature search was performed in Medline (OvidSP), Pubmed Publisher, EMBASE, Google Scholar and the Cochrane Controlled Clinical Trials Register. Two complementary search strategies were used for optimal article retrieval. The first strategy, focusing on the three atopic disorders, combined the following items: “Child” AND “Epidemiology” AND “Asthma” AND “Allergic rhinitis” AND “Eczema”. The second strategy, focusing on ISAAC studies, used additional items and different Boolean operators: “Child” AND “Epidemiology” AND (“Asthma” OR “Allergic rhinitis” OR “Eczema”) AND (“ISAAC” OR “International Study of Asthma and Allergies”).

The full search strategies can be found in S1 Appendix. Since ISAAC started in 1991, only full-text articles published after 1991 were considered; there was no language restriction. The search was completed on February 2, 2015. A reference check was made on all articles finally included.

### Study selection

Studies (n>100) with a cross-sectional or cohort design, including youngsters aged 0–18 years, recruited in the open population (e.g. schools) were included. Studies using the ISAAC questionnaire, performed by both official and non-official ISAAC research groups, were included if the studies presented data on the prevalence of all three atopic diseases and their interrelationships.

One reviewer (EA) commenced the selection of studies, initially based on title and abstract. To check for any missed inclusions by the first reviewer, a random selection of 50% of the articles was independently checked by second reviewers (DP, JW, AB, NR, HM). This check showed that the first reviewer did not exclude any potentially relevant articles.

Of the abstracts selected, the full-text articles were retrieved. Two reviewers (EA and NR) independently performed the full-text selection using a standardized form based on the above-mentioned inclusion criteria. Studies were excluded if they only presented aggregated worldwide data, or when double inclusion of the data could not be ruled out. Disagreement was resolved in a consensus meeting or with the help of a third independent reviewer (DP). Authors of the studies were contacted regarding missing data.

### Quality assessment

To minimize the risk of information bias, the quality of the included studies was assessed by two independent reviewers (DP and AB). Disagreement was resolved in a consensus meeting.

ISAAC used the same standardized method in ISAAC phase 1 and 3. Methodological differences between these phases were studied [11] and it was concluded that the ISAAC methodology was replicated to a high degree by the majority of the study centers. This showed that the ISAAC protocol is robust and that working in accordance with this protocol implied substantial generalizability. Any important violations of this protocol were, therefore, identified in order to assess quality (Table 1).

**Table 1:** Study characteristics and quality assessment.

**Article**	ISAAC	No. analyzed*	Age range (years)	% Males	Response rate	Study year	Continent	English Question.	Violation protocols†
Ait-Khaled 2007 [13] ‡	Yes	4,123	13–14	58.3	99.1	1996–1999	Africa	No	None
Asher 2001 [14]	Yes	18,569/19,023	6-7/13-14	50.6/46.7	91.2/92.6	1992–1993	Oceania	Yes	None
Austin 1999 [15]	Yes	27,507	12–14	49.2	85.9	1995	Europe	Yes	6
Bröms 2013 [16]	No	4,886	1–6	50,7	67,5	2002	Europe	No	1, 2, 3, 4, 6
Cibella 2011 [17]	No	2,150	10–17	49.2	87.8	2005–2006	Europe	No	2, 3, 6, 7
Civelek 2010 [18]	No	5,664	9–11	50.9	91.3	2005–2006	Europe	No	3
Duggan 2012 [19]	No	1,474/1,535	6–9	47.3/47.9	74.8/76.2	2002/2007	Europe	Yes	3, 6, 7
Eder 1998 [20]	Yes	3,672/3,371	6-8/12-15	51.0/51.0	88.2/85.1	1995	Europe	No	3, 6
Falade 2004 [21]	Yes	1,312	6–7	46.8	73.3	1995	Africa	Yes	6, 7
Futamura 2011 [22]	No	27,389	6–14	47.2	74.4	2005	Asia	No	3, 6
Ghaffari 2012 [23]	No	1,818	7–12	65.0	91.0	2010	Asia	No	3, 4, 5, 7
Hailu 2003 [24]	No	3,365	13–14	40.1	98.4	1997	Africa	No	None
Hong 2012 [25]	No	31,201	0–13	51.1	82.1	2010	Asia	No	3, 4, 5, 6
Janahi 2006 [26]	No	3,283	6–14	52.3	93.8	2003–2004	Asia	No	3, 5
Lamnisos 2013 [27]	No	4,569/5,587	7-8/13-14	50,0/49,8	56,8/68,1	2007–2009	Europe	No	2, 3, 6
Liao 2005 [28]	No	7,040	6–8	51.5	89.4	2002	Asia	No	3, 6
Liao 2009 [29]	No	4,622	6–8	53.2	79.1	2007	Asia	No	3, 6
Mallol 2012 [30]	Yes	388,453/796,368	6-7/13-14	50.3/49.3	-	2000–2003	World	Yes/No	6
Manning 1997 [31]	Yes	3,148	13–14	46.0	92.1	1995	Europe	Yes	None
Marinho 2007 [32]	No	815	5	54.7	67.3	1998–2002?	Europe	Yes	1, 2, 3, 5, 6, 7
Martin Fernández 2004 [33]	Yes	3,018	13–14	51.4	100.0	<2002?	Europe	No	None
Musharrafieh 2009 [34]	No	3,115	13–14	48.6	-	2005	Asia	No	2, 6
Nwaru 2013 [35]	No	2,448	5	53.0	93.0	2001–2009	Europe	No	1, 2, 3, 7
Rahimi Rad 2007 [36]	No	3,000	13–14	50.0	98.3	2002–2003	Asia	No	None
Rahimi Rad 2008 [37]	No	2,999	6–7	48.4	94.4	2002–2003	Asia	No	7
Remes 1998 [38]	Yes	11,607	13–14	49.5	96.5	1994–1995	Europe	No	None
Robertson 1998 [39]	Yes	10,914/12,280	6-7/13-14	-	84.3/93.9	1993–1994	Oceania	Yes	6
Škvorc 2014 [40]	No	3,106	7–9	50,4	96,9	2005	Europe	No	3, 4
Wördemann 2006 [41]	No	398	5–13	48.0	100.0	2003–2004	North America	No	3, 4, 5, 7
Yao 2011 [42]	No	5,155	4–18	48.9	94.9	2007	Asia	No	3, 4, 5
Ziyab 2014 [43]	No	1,345	10	50,7	92,4	1999	Europe	Yes	1, 2, 3, 5, 7

* Number of patients available for analysis

^**†**^ 1) Recruitment at schools; 2) All schools or randomly selected; 3) Age groups 6–7 and 13–14 years; 4) Use of validated questionnaires; 5) questionnaires completed by parents (< 12 years old) or by adolescents themselves (≥ 12 years old); 6) Participation >90%; 7) N ≥3000

^‡^ Only two centers were included (Benslimane, Morocco; Conakry, Guinea.

The present review includes only those articles that used the ISAAC questionnaires. This questionnaire has been translated into various languages by regional coordinators of ISAAC, using a consistent protocol that was evaluated by Ellwood et al. [12]. Use of a validated questionnaire was also considered an important quality item and was part of the quality assessment (Table 1).

The above mentioned violations and the use of the original questionnaire or not could potentially influence the comparability of ISAAC and non-ISAAC studies. For this reason we performed a meta-regression analyses in order to evaluate if these violation would influence our outcomes (prevalence and RR).

### Data extraction

Two reviewers (EA/JW and DP) independently extracted data from the included studies. A standardized digital form was used to record study design, participants, official ISAAC study or not, and outcome measures. In view of the outcome measures, the total number of participants and the number of participants with asthma (As), eczema (Ec), allergic rhinitis (AR) and of As+Ec, As+AR, Ec+ AR and As+Ec+AR were extracted. These numbers were then entered in the Review Manager (RevMan) Computer program (Version 5.1. Copenhagen: The Nordic Cochrane Centre, The Cochrane Collaboration, 2011). This program provides risk ratios, 95% confidence intervals (CI) (using the Mantel-Haenszel test and random effects models) and the weight of every study. The extraction was limited to current symptoms (past 12 months) and data collected by written questionnaires. Study characteristics regarding gender, age, continent, validated (English) questionnaires, ISAAC/non-ISAAC study, number of participants, response rate, study period and ISAAC protocol violations were also collected. Data entry was additionally checked by two independent reviewers (AB, JW).

### Statistical analyses

In order to calculate the mean prevalences, the studies were weighted for their number of participants. Risk ratios (RR) calculated by RevMan describe the risk of having two different atopic disorders when a child is known with one disorder. For example, if the RR for asthma is four, this would mean that a child with asthma has a fourfold risk of reporting eczema and allergic rhinitis in contrast to a child without asthma. Heterogeneity (I^2^) was assessed using a random effects model.

For the study characteristics of this meta-analysis, a mixed-effects model was used for natural logarithm of the calculated RR for asthma, eczema and allergic rhinitis and for the prevalence of asthma, eczema, allergic rhinitis and having all three disorders. Initial models for these seven responses contained all covariates of interest as fixed effects: percentage of males, age, continent, ISAAC/non-ISAAC, number of participants, response rate, study period and the use of validated English questionnaires. The latter was chosen to explore the influence of using translations on the RR. Since not all studies provided data on the percentage of male participants and the response rate, and both variables did not have significant parameters in the complete case analysis, both variables were excluded from the models in order to be able to use all 57 studies for the meta regression. Some influential centers were removed from initial and final models using traditional measures: standardized residuals, DFFITS values, Cook's distance and hat values. All calculations were conducted in R with the metafor package (Wolfgang Viechtbauer (2010)). A p value of 0.01 was considered the limit of significance because of multiple testing (Bonferroni correction).

## Results

### Identification and selection of the literature

**Fig 1:**
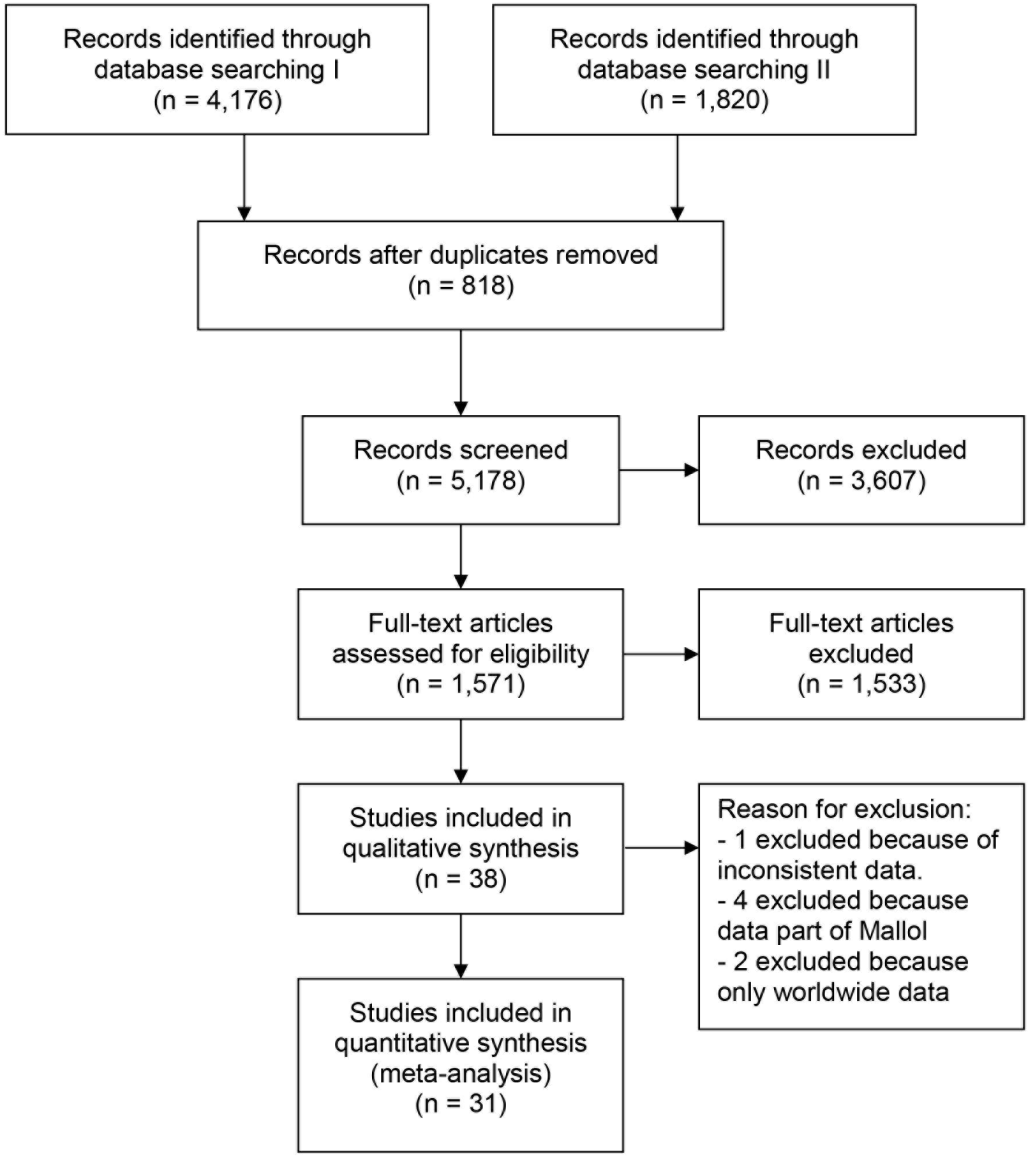
Flow diagram for selection of studies identified in the systematic review.

The combined search strategies resulted in 5,178 original abstracts. No articles were excluded because of language barriers but the majority (n = 3,607; 69.7%) did not meet the inclusion criteria, mainly because these articles did not present data on all three disorders or because ISAAC questionnaires were not used. We retrieved 1,571 full-text articles for detailed evaluation. Of these, another 1,533 studies were not included, mainly because the studies did not use ISAAC questionnaires or because these articles did not present data on all three disorders. Finally 38 studies were initially included in this review for further analysis [2, 13–49] (Fig 1).

### Description and final selection of studies

The ISAAC Phase 3 synthesis presented by Mallol et al. [30] covers a large number of surveyed children (n = 1,184,821). Four of the included ISAAC studies [45–48] were excluded because it is assumed that the data from these studies were already included by Mallol et al. [30]. The data presented by Song et al. [49] showed internal inconsistency and was therefore excluded. Furthermore, articles that only presented worldwide data (n = 2) [44, 50] were not used for the final analysis.

Finally, data from 31 studies were used, covering a large number of surveyed children (n = 1,430,329) in 102 different countries. Table 1 presents descriptive characteristics of the studies, including the results of the quality assessment. All officially acknowledged ISAAC studies, with the exception of one [38], used the same definition for asthma, eczema and allergic rhinitis. Non-ISAAC studies varied considerably in the definitions they used for the disorders.

### Overall and regional difference in prevalence of atopic manifestations

**Fig 2:**
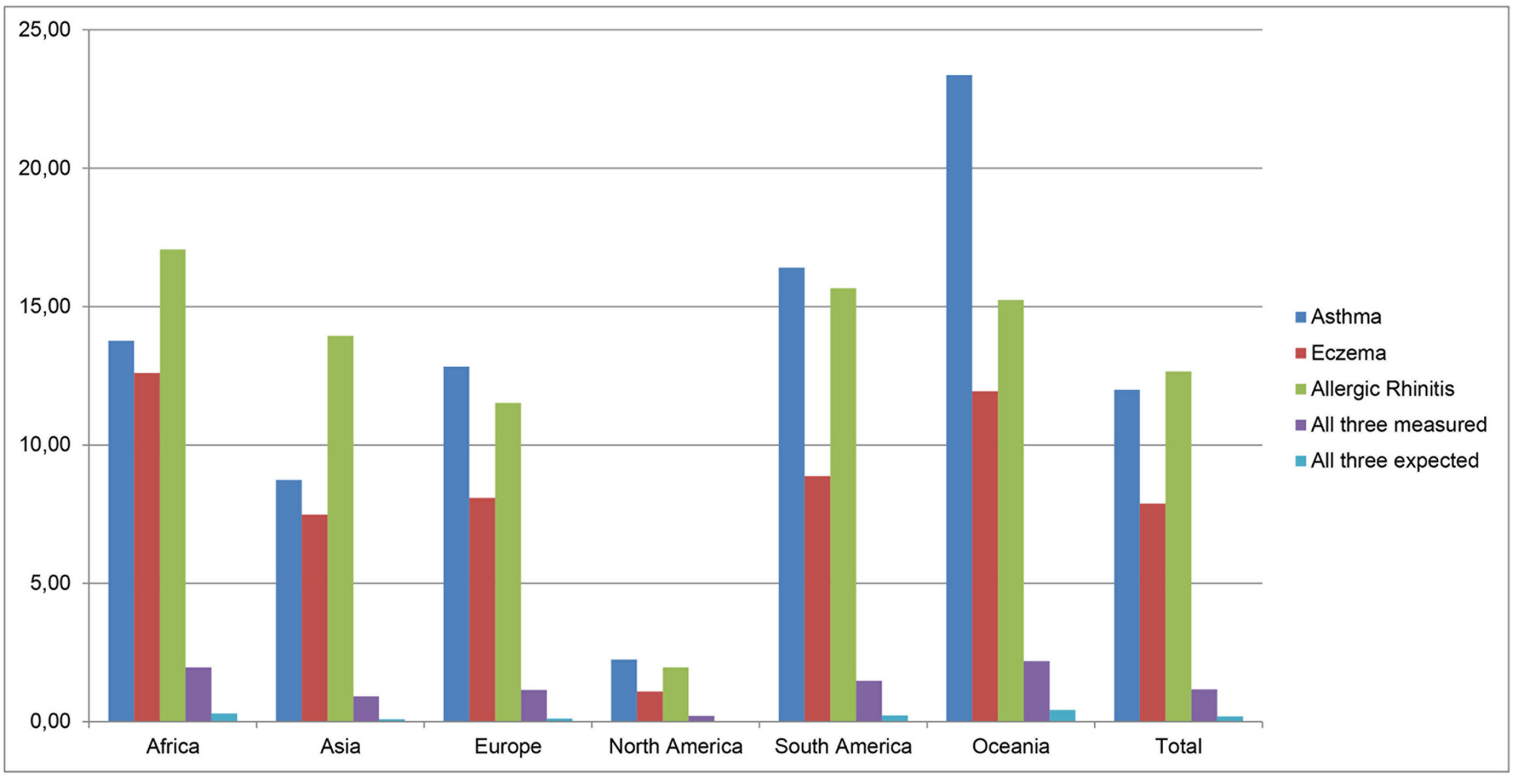
Prevalence (%) of the atopic disorders per continent.

The calculated worldwide prevalence for asthma, eczema and allergic rhinitis for children in the open population is 12.00% (95% CI: 11.99–12.00), 7.89% (95% CI: 7.88–7.89) and 12.66% (95% CI: 12.65–12.67), respectively. Fig 2 shows the prevalence per continent. None of the continents significantly influenced the worldwide prevalence of any one of the atopic disorders, neither did percentage of males, ISAAC/non-ISAAC, number of participants and the use of validated English questionnaires. There were significant negative associations between age and prevalence of eczema and between study period and prevalence of asthma. The worldwide observed prevalence of having all three diseases is 1.17% (95% CI: 1.17–1.17) and was not influenced by the above mentioned factors. If there would be no interrelationship at all between the three disorders, the expected worldwide prevalence of having all three disorders is only 0.12% (12.00%*7.89%*12.66%)). In the present review, the observed prevalence is 9.8 times higher than could be expected by chance, suggesting a close relationship between these disorders in children. It is remarkable that the prevalence of ‘all three expected’ is relatively consistent between the six continents (Fig 2).

### Interrelationship between the atopic manifestations

**Fig 3:**
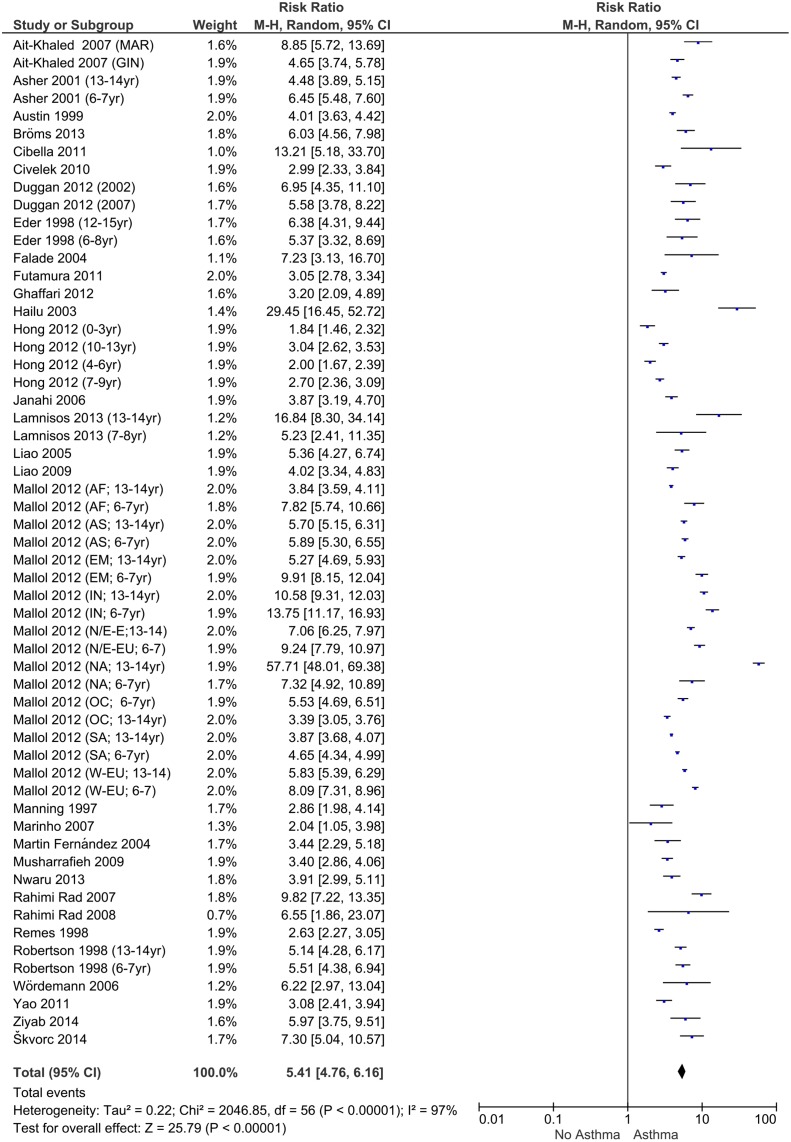
Forest plot of risk ratios for asthma.

**Fig 4:**
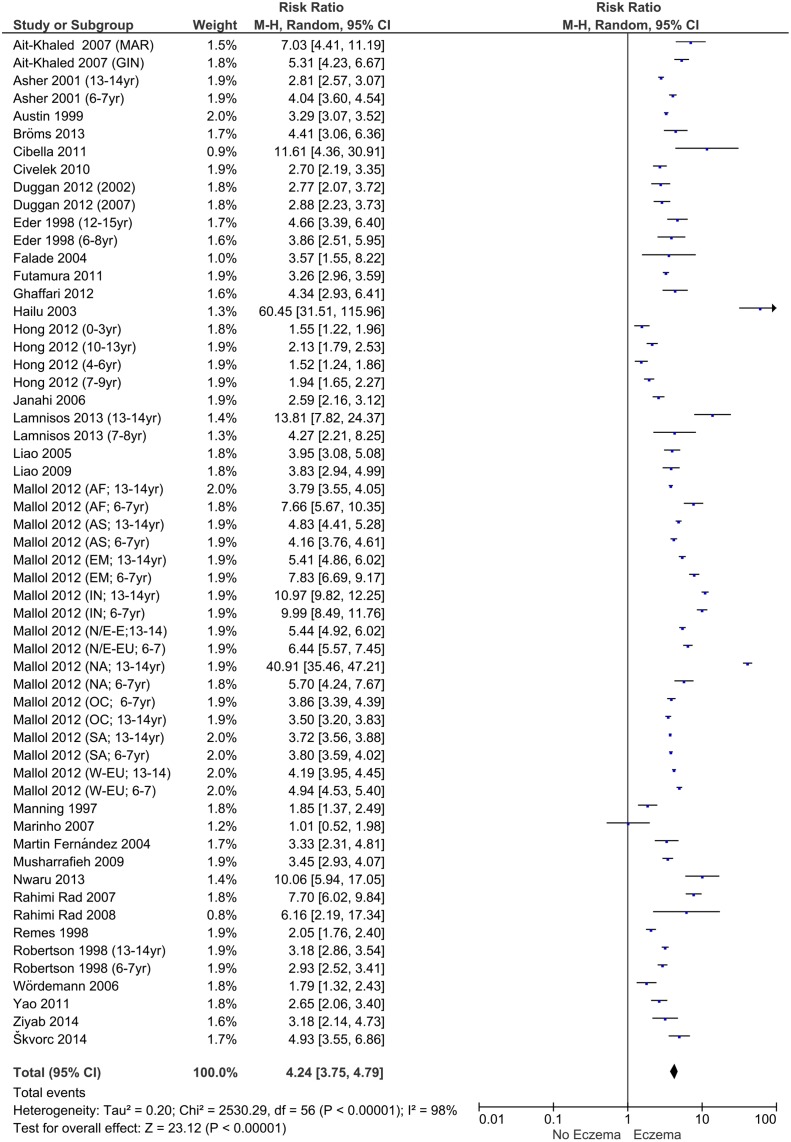
Forest plot of risk ratios for eczema.

**Fig 5:**
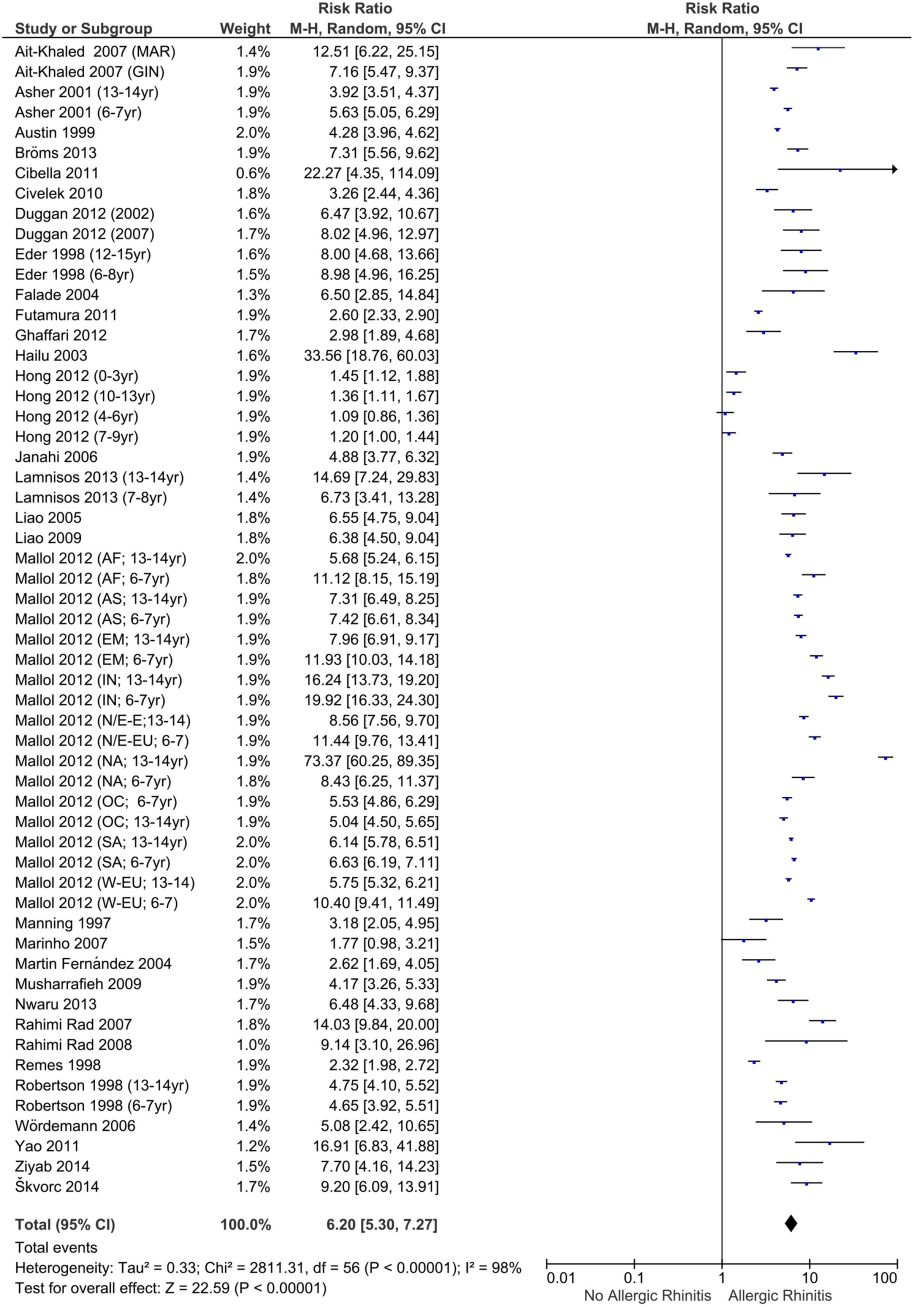
Forest plot of risk ratios for allergic rhinitis.

Calculated RR for children with asthma, eczema and allergic rhinitis are presented in the Forest plots (Figs 3–5). If possible, the Forest plots provide a subdivision per article by continent and age. The overall RR for patients having asthma to also suffer from eczema and rhinitis is 5.41 (95% CI:4.76–6.16). For patients with eczema the RR is 4.24 (95% CI: 3.75–4.79) and for allergic rhinitis the RR is 6.20 (95% CI: 5.30–7.27). These risk ratios show a clear relationship of the three disorders. Additional analyses to examine whether RRs were influenced by covariates (percentage of males, age, continent, official ISAAC/non-ISAAC study, number of participants, response rate, study period and the use of validated English questionnaires) showed no significant effect on the calculated RR.

There is substantial heterogeneity (I^2^ = 97–98%) between these studies. Subanalyses performed for different subgroups (percentage of males, age, continent, ISAAC/non-ISAAC, number of participants, response rate, study period) showed no major change in heterogeneity.

## Discussion

A comprehensive literature search retrieved data from 102 different countries, making this one of the largest meta-analysis of asthma, eczema and allergic rhinitis ever conducted. The calculated worldwide prevalence for asthma, eczema and allergic rhinitis for children in the open population is 12.0%, 7.9% and 12.7%, respectively. Overall this prevalence is higher than that presented earlier by Mallol et al [30]. None of the individual continents had a significant influenced on the worldwide prevalence of one of the atopic disorders.

In this review, the observed prevalence of having all three disorders is 1.17% (95% CI: 1.17–1.18). This co-occurrence is substantially higher than could be expected by chance, based on the individual prevalence of each disorder (0.12%). This supports the hypothesis that there could be a fourth distinct group of children with all three disorders. A new and different way of looking at the interrelationships is by calculating RRs; the RRs presented in this review, describe the risk of having the other two atopic disorders when a child is known with one disorder. The RRs ranged from 4.24–6.20 and were not significantly influenced by any of the confounders investigated. Since all RR were > 1, this implies that the observed co-occurrence is not based on chance, but suggest a clear relationship between the disorders. Remarkably, the RR of eczema is low compared with the other two disorders; this might be because we used prevalence data based on having complaints in the past 12 months and not on lifetime prevalences. On average, eczema is seen in children at a younger age than those studied in this review, thereby resulting in a lower RR. This study also showed a significant decline in the prevalence of asthma when a child becomes older.

The wide variation in the prevalence of atopic diseases [1–4] has received considerable attention. Possible causes of these variations include (amongst others): genetics, use of paracetamol, use of antibiotics, breastfeeding, diet, body mass index, living in a rural area, and air pollution. However, none of these proposed factors fully explains this wide variation. Remarkably, when looking at the prevalence of having all three disorders, this wide variation does not occur to the same extent. In the present study, the limited degree of overlap between the three conditions (1.17%) was very similar to that reported by others [30, 50]. Asher et al. [44] even showed that this overlap has been relatively consistent over a period of seven years; for 6–7 year olds this overlap increased from 0.8% to 1.0% and for the 13–14 year olds the overlap increased from 1.1% to 1.3%. This consistency in prevalence also suggests that a fourth group of children with atopic disorders might exist. In addition to the three regularly described groups of children with asthma, eczema or allergic rhinitis, there seems to be a fourth distinct group of children with all three disorders, that may show distinct characteristics regarding severity, causes, treatment or prognosis.

We suggest to add another chapter to the already impressive ISAAC study, focusing on this potentially distinct fourth group of children with all three manifestations. Is this group distinctive due to severity of symptoms? Does this group have a different genotype? Does this group need a different pharmacological approach? Does this group have a different prognosis? Which factors influence this group?

This meta-analysis has some limitations. First, one reviewer selected the studies based on title and abstract. Despite a random check of 50% of the retrieved articles showing concordance, we assumed that no relevant articles were missed. However, the full-text selection was done by two independent reviewers. In our review there was no limitation for any language and (where possible) authors were contacted for missing data.

When conducting a large multicenter international cross-sectional study, there is always a risk of potential limitations. Clear examples include language problems, cultural differences, environmental aspects, different healthcare systems, etc. Either an overestimation or an underestimation might be found. Another concern is the possible overestimation of the prevalence of the three atopic diseases. Although the questionnaires asked about symptoms, the symptoms could well be attributable to other diseases; this concern is shared by others [28, 46, 48, 51]. Furthermore, Cane et al.[52] showed that the conceptual understanding of ‘wheeze’ differs between reporting parents and epidemiology definitions. Finally, different research groups used different definitions for the atopic disorders; this could have influenced our results.

The high level of heterogeneity that we found suggests that the included studies differ significantly from each other. However, this can be explained by the large number of participants in each study. Because the studies have such large populations, the CIs will be very small. Even small differences will result in statistical heterogeneity, but not in clinical heterogeneity.

This meta analyses supports the hypothesis that these three atopic disorders are clearly related. A biological plausible pathway for these relationships can be found in the atopic march theory. However, the obtained data in this meta-analysis does not allow to quantify the effect of this atopic march theory. This is due to two limitations. The first limitation relates to the cross sectional methods used. We have no follow-up data available for an individual child. The second one is that we limited our data inclusion to symptoms within the previous year (year prevalence). Using year-prevalences instead of life-time prevalences could result in an underestimation of the prevalences. Atopic dermatitis often goes into a clinical remission, but the atopic phenotype persists. The same applies to asthma. For example, when establishing the prevalence at the age of e.g. 12 years, the child may answer no, but in fact might still have an atopic phenotype.

## Conclusions

We studied the prevalence and interrelationships between asthma, allergic rhinitis and eczema in children using data obtained from ISAAC questionnaires. The interrelationships were studied using risk ratios, adjusted for potential confounders. Our meta-analysis has shown that the prevalence of children with a co-occurrence of asthma, eczema and allergic rhinitis was low, but significantly higher than could be expected by chance. The prevalence of having all three atopic disorders was remarkably consistent in all continents. This study supports the hypothesis that there might be a forth distinct group of children with all three disorders, in contrast to the traditional classification of children with asthma or allergic rhinitis or atopic eczema. Researchers and clinicians might need to consider this forth group as a separate group of children with their own characteristics.

## Supporting Information

S1 Appendix Search strings used for literature search. (DOCX)

S2 Appendix PRISMA 2009 Checklist. (DOC)

## References

[1] Strachan D, Sibbald B, Weiland S, Ait-Khaled N, Anabwani G, Anderson HR, et al Worldwide variations in prevalence of symptoms of allergic rhinoconjunctivitis in children: the International Study of Asthma and Allergies in Childhood (ISAAC). Pediatr Allergy Immunol. 1997;8(4):161–76. . 9553981 10.1111/j.1399-3038.1997.tb00156.x

[2] The International Study of Asthma and Allergies in Childhood (ISAAC) Steering Committee. Worldwide variation in prevalence of symptoms of asthma, allergic rhinoconjunctivitis, and atopic eczema: ISAAC. Lancet. 1998;351(9111):1225–32. . 9643741

[3] The International Study of Asthma and Allergies in Childhood (ISAAC) Steering Committee. Worldwide variations in the prevalence of asthma symptoms: the International Study of Asthma and Allergies in Childhood (ISAAC). Eur Respir J. 1998;12(2):315–35. . 9727780 10.1183/09031936.98.12020315

[4] Williams H, Robertson C, Stewart A, Ait-Khaled N, Anabwani G, Anderson R, et al Worldwide variations in the prevalence of symptoms of atopic eczema in the International Study of Asthma and Allergies in Childhood. J Allergy Clin Immunol. 1999;103(1 Pt 1):125–38. . 9893196 10.1016/s0091-6749(99)70536-1

[5] Burgess JA, Lowe AJ, Matheson MC, Varigos G, Abramson MJ, Dharmage SC. Does eczema lead to asthma? J Asthma. 2009;46(5):429–36. 10.1080/02770900902846356 19544160

[6] Duczmal E, Breborowicz A, Duczmal T. Allergic march in childhood. Postepy Dermatol Alergol. 2010;27(4):231–7.

[7] Sun HL, Yeh CJ, Ku MS, Lue KH. Coexistence of allergic diseases: Patterns and frequencies. Allergy Asthma Proc. 2012;33(1):e1–e4. 10.2500/aap.2012.33.3506 22370527

[8] Asher MI, Keil U, Anderson HR, Beasley R, Crane J, Martinez F, et al International Study of Asthma and Allergies in Childhood (ISAAC): rationale and methods. Eur Respir J. 1995;8(3):483–91. . 7789502 10.1183/09031936.95.08030483

[9] Ellwood P, Asher MI, Beasley R, Clayton TO, Stewart AW, Committee IS. The international study of asthma and allergies in childhood (ISAAC): phase three rationale and methods. Int J Tuberc Lung Dis. 2005;9(1):10–6. . 15675544

[10] Weiland SK, Bjorksten B, Brunekreef B, Cookson WO, von Mutius E, Strachan DP, et al Phase II of the International Study of Asthma and Allergies in Childhood (ISAAC II): rationale and methods. Eur Respir J. 2004;24(3):406–12. . 15358699 10.1183/09031936.04.00090303

[11] Ellwood P, Asher MI, Stewart AW, Ait-Khaled N, Mallol J, Strachan D, et al The challenges of replicating the methodology between Phases I and III of the ISAAC programme. Int J Tuberc Lung Dis. 2012;16(5):687–93. 10.5588/ijtld.11.0226 22507933

[12] Ellwood P, Williams H, Ait-Khaled N, Bjorksten B, Robertson C, Group IPIS. Translation of questions: the International Study of Asthma and Allergies in Childhood (ISAAC) experience. Int J Tuberc Lung Dis. 2009;13(9):1174–82. . 19723410

[13] Ait-Khaled N, Odhiambo J, Pearce N, Adjoh KS, Maesano IA, Benhabyles B, et al Prevalence of symptoms of asthma, rhinitis and eczema in 13- to 14-year-old children in Africa: The International Study of Asthma and Allergies in Childhood Phase III. Allergy Eur J Allergy Clin Immunol. 2007;62(3):247–58. 10.1111/j.1398-9995.2007.01325.x 17298341

[14] Asher MI, Barry D, Clayton T, Crane J, D'Souza W, Ellwood P, et al The burden of symptoms of asthma, allergic rhinoconjunctivitis and atopic eczema in children and adolescents in six New Zealand centres: ISAAC Phase One. N Z Med J. 2001;114(1128):114–20. 11346157

[15] Austin JB, Kaur B, Anderson HR, Burr M, Harkins LS, Strachan DP, et al Hay fever, eczema, and wheeze: A nationwide UK study (ISAAC, international study of asthma and allergies in childhood). Arch Dis Child. 1999;81(3):225–30. 10451395 10.1136/adc.81.3.225 PMC1718047

[16] Broms K, Norback D, Eriksson M, Sundelin C, Svardsudd K. Prevalence and co-occurrence of parentally reported possible asthma and allergic manifestations in pre-school children. BMC Public Health. 2013;13:764 10.1186/1471-2458-13-764 23953349 PMC3765705

[17] Cibella F, Cuttitta G, La Grutta S, Melis MR, Lospalluti ML, Uasuf CG, et al Proportional Venn diagram and determinants of allergic respiratory diseases in Italian adolescents. Pediatr Allergy Immunol. 2011;22(1 Pt 1):60–8. 10.1111/j.1399-3038.2010.01097.x . 20825572

[18] Civelek E, Cakir B, Boz AB, Yuksel H, Orhan F, Uner A, et al Extent and burden of allergic diseases in elementary schoolchildren: A national multicenter study. J Invest Allergol Clin Immunol. 2010;20(4):280–8. 20815305

[19] Duggan EM, Sturley J, Fitzgerald AP, Perry IJ, Hourihane JO. The 2002–2007 trends of prevalence of asthma, allergic rhinitis and eczema in Irish schoolchildren. Pediatr Allergy Immunol. 2012;23(5):464–71. 10.1111/j.1399-3038.2012.01291.x 22435792

[20] Eder W, Gamper A, Oberfeld G, Riedler J. Prevalence and severity of bronchial asthma, allergic rhinitis and atopic dermatitis in Salzburg school children. Wien Klin Wochenschr. 1998;110(19):669–77. 9823620

[21] Falade AG, Olawuyi JF, Osinusi K, Onadeko BO. Prevalence and severity of symptoms of asthma, allergic rhinoconjunctivitis, and atopic eczema in 6- to 7-year-old Nigerian primary school children: The International Study of Asthma and Allergies in Childhood. Med Princ Pract. 2004;13(1):20–5. 14657614 10.1159/000074046

[22] Futamura M, Ohya Y, Akashi M, Adachi Y, Odajima H, Akiyama K, et al Age-related prevalence of allergic diseases in Tokyo schoolchildren. Allergol Int. 2011;60(4):509–15. 10.2332/allergolint.10-OA-0293 21778812

[23] Ghaffari J, Mohammadzadeh I, Khalilian A, Rafatpanah H, Mohammadjafari H, Davoudi A. Prevalence of asthma, allergic rhinitis and eczema in elementary schools in sari (Iran). Caspian J Int Med. 2012;3(1):372–6. PMC4600135 26557289

[24] Hailu S, Tessema T, Silverman M. Prevalence of symptoms of asthma and allergies in schoolchildren in Gondar town and its vicinity, Northwest Ethiopia. Pediatr Pulmonol. 2003;35(6):427–32. 12746938 10.1002/ppul.10215

[25] Hong S, Son DK, Lim WR, Kim SH, Kim H, Yum HY, et al The prevalence of atopic dermatitis, asthma, and allergic rhinitis and the comorbidity of allergic diseases in children. Environ Health Toxicol. 2012;27:e2012006 10.5620/eht.2012.27.e2012006 22359737 PMC3282234

[26] Janahi IA, Bener A, Bush A. Prevalence of asthma among Qatari schoolchildren: International study of asthma and allergies in childhood, Qatar. Pediatr Pulmonol. 2006;41(1):80–6. 16283628 10.1002/ppul.20331

[27] Lamnisos D, Moustaki M, Kolokotroni O, Koksoy H, Faiz M, Arifoglu K, et al Prevalence of asthma and allergies in children from the Greek-Cypriot and Turkish-Cypriot communities in Cyprus: a bi-communal cross-sectional study. BMC Public Health. 2013;13:585 10.1186/1471-2458-13-585 23767800 PMC3698153

[28] Liao MF, Huang JL, Chiang LC, Wang FY, Chen CY. Prevalence of asthma, rhinitis, and eczema from ISAAC survey of schoolchildren in Central Taiwan. J Asthma. 2005;42(10):833–7. 16393720 10.1080/02770900500369892

[29] Liao MF, Liao MN, Lin SN, Chen JY, Huang JL. Prevalence of allergic diseases of schoolchildren in central Taiwan: From ISAAC surveys 5 years apart. J Asthma. 2009;46(6):541–5. 10.1080/02770900902795546 19657892

[30] Mallol J, Crane J, von Mutius E, Odhiambo J, Keil U, Stewart A. The International Study of Asthma and Allergies in Childhood (ISAAC) Phase Three: A global synthesis. Allergol Immunopathol. 2012;41(2):73–85. 10.1016/j.aller.2012.03.001 22771150

[31] Manning PJ, Curran K, Kirby B, Taylor MR, Clancy L. Asthma, hay fever and eczema in Irish teenagers (ISAAC protocol). Ir Med J. 1997;90(3):110–2. 9183097

[32] Marinho S, Simpson A, Lowe L, Kissen P, Murray C, Custovic A. Rhinoconjunctivitis in 5-year-old children: A population-based birth cohort study. Allergy Eur J Allergy Clin Immunol. 2007;62(4):385–93. 10.1111/j.1398-9995.2006.01294.x 17362249

[33] Martin Fernandez-Mayoralas D, Martin Caballero JM, Garcia-Marcos Alvarez L. Association between atopic dermatitis, allergic rhinitis and asthma in schoolchildren aged 13–14 years old. An Pediatr. 2004;60(3):236–42. 10.1016/s1695-4033(04)78257-0 14987514

[34] Musharrafieh U, Al-Sahab B, Zaitoun F, El-Hajj MA, Ramadan F, Tamim H. Prevalence of asthma, allergic rhinitis and eczema among lebanese adolescents. J Asthma. 2009;46(4):382–7. 10.1080/02770900902777775 19484674

[35] Nwaru BI, Takkinen HM, Niemela O, Kaila M, Erkkola M, Ahonen S, et al Timing of infant feeding in relation to childhood asthma and allergic diseases. J Allergy Clin Immunol. 2013;131(1):78–86. 10.1016/j.jaci.2012.10.028 23182171

[36] Rahimi Rad MH, Hejazi ME, Behrouzian R. Asthma and other allergic diseases in 13-14-year-old schoolchildren in Urmia: An ISAAC study. East Mediterr Health J. 2007;13(5):1005–16. 18290392 10.26719/2007.13.5.1005

[37] Rahimi Rad MH, Hamzezadeh A. Allergic disease in 6–7-year-old schoolchildren in Urmia, Islamic Republic of Iran. East Mediterr Health J. 2008;14(5):1044–53. 19161076

[38] Remes ST, Korppi M, Kajosaari M, Koivikko A, Soininen L, Pekkanen J. Prevalence of allergic rhinitis and atopic dermatitis among children in four regions of Finland. Allergy Eur J Allergy Clin Immunol. 1998;53(7):682–9. 10.1111/j.1398-9995.1998.tb03954.x 9700037

[39] Robertson CF, Dalton MF, Peat JK, Haby MM, Bauman A, Kennedy JD, et al Asthma and other atopic diseases in Australian children. Australian arm of the International Study of Asthma and Allergy in Childhood. Med J Aust. 1998;168(9):434–8. 9612454

[40] Skvorc HM, Plavec D, Munivrana S, Skvorc M, Nogalo B, Turkalj M. [The prevalence of symptoms of allergic diseases among younger school children in Medimurje County (ISAAC Phase I)] Croatian. Lijec Vjesn. 2014;136(3–4):73–8. . 24988740

[41] Wordemann M, Polman K, Diaz RJ, Menocal Heredia LT, Madurga AMC, Sague KA, et al The challenge of diagnosing atopic diseases: Outcomes in Cuban children depend on definition and methodology. Allergy Eur J Allergy Clin Immunol. 2006;61(9):1125–31. 10.1111/j.1398-9995.2006.01129.x 16918517

[42] Yao TC, Ou LS, Yeh KW, Lee WI, Chen LC, Huang JL. Associations of age, gender, and BMI with prevalence of allergic diseases in children: PATCH study. J Asthma. 2011;48(5):503–10. 10.3109/02770903.2011.576743 21599561

[43] Ziyab AH, Karmaus W, Zhang H, Holloway JW, Steck SE, Ewart S, et al Allergic sensitization and filaggrin variants predispose to the comorbidity of eczema, asthma, and rhinitis: Results from the Isle of Wight birth cohort. Clin Exp Allergy. 2014;44(9):1170–8. 10.1111/cea.12321 24708301 PMC4140962

[44] Asher MI, Stewart AW, Wong G, Strachan DP, Garcia-Marcos L, Anderson HR. Changes over time in the relationship between symptoms of asthma, rhinoconjunctivitis and eczema: A global perspective from the International Study of Asthma and Allergies in Childhood (ISAAC). Allergol Immunopathol. 2012;40(5):267–74. 10.1016/j.aller.2011.11.004 22297190

[45] Batlles-Garrido J, Torres-Borrego J, Rubi-Ruiz T, Bonillo-Perales A, Gonzalez-Jimenez Y, Momblan-De Cabo J, et al Prevalence and factors linked to allergic rhinitis in 10 and 11-year-old children in Almeria. Isaac Phase II, Spain. Allergol Immunopathol. 2010;38(3):135–41. 10.1016/j.aller.2009.09.005 20462685

[46] Clausen M, Kristjansson S, Haraldsson A, Bjorksten B. High prevalence of allergic diseases and sensitization in a low allergen country. Acta Paediatr Int J Paediatr. 2008;97(9):1216–20. 10.1111/j.1651-2227.2008.00887.x 18631343

[47] Kao CC, Huang JL, Ou LS, See LC. The prevalence, severity and seasonal variations of asthma, rhinitis and eczema in Taiwanese schoolchildren. Pediatr Allergy Immunol. 2005;16(5):408–15. 16101933 10.1111/j.1399-3038.2005.00268.x

[48] Riedi CA, Rosario NA, Ribas LFO, Backes AS, Kleiniibing GF, Popija M, et al Increase in prevalence of rhinoconjunctivitis but not asthma and atopic eczema in teenagers. J Investig Allergol Clin Immunol. 2005;15(3):183–8. . 16261954

[49] Song N, Mohammed S, Zhang J, Wu J, Fu C, Hao S, et al Prevalence, severity and risk factors of asthma, rhinitis and eczema in a large group of Chinese schoolchildren. J Asthma. 2014;51(3):232–42. 10.3109/02770903.2013.867973 24303994

[50] The International Study of Asthma and Allergies in Childhood (ISAAC) Steering Committee. Worldwide variation in prevalence of symptoms of asthma, allergic rhinoconjunctivitis, and atopic eczema: ISAAC. Lancet. 1998;351(9111):1225–32. . 9643741

[51] Brescianini S, Brunetto B, Iacovacci P, D'Ippolito C, Alberti G, Schirru MA, et al Prevalence of self-perceived allergic diseases and risk factors in Italian adolescents. Pediatr Allergy Immunol. 2009;20(6):578–84. 10.1111/j.1399-3038.2008.00793.x 18710432

[52] Cane RS, Ranganathan SC, McKenzie SA. What do parents of wheezy children understand by "wheeze"? Arch Dis Child. 2000;82(4):327–32. . 10735844 10.1136/adc.82.4.327 PMC1718271

