# Supplementary material for: Interrelationships between Atopic Disorders in Children: A Meta-Analysis Based on ISAAC Questionnaires
Source: PLoS One. 2015 Jul 2;10(7):e0131869. doi: 10.1371/journal.pone.0131869 (PMC4489894; doi:10.1371/journal.pone.0131869)
Supplement: S1 Appendix — (DOCX) [file pone.0131869.s001.docx]

***S1 Appendix***

***Search string ONE contained the following specific terms:***

***For EMBASE:***

(asthma/exp OR wheezing/de OR (asthma* OR wheez* OR hyperresponsiv* OR hypersensit* OR (hyper NEXT/1 (responsiv* OR sensitiv*))):ab,ti) AND (eczema/de OR 'atopic dermatitis'/de OR (eczem* OR (atopic NEAR/3 dermatit*)):ab,ti) AND (rhinitis/exp OR conjunctivitis/exp OR (rhinitis* OR rhinoconjunctivit* OR conjunctivit* OR (Pollen NEAR/3 Allerg*) OR Pollinos* OR ((hay) NEXT/1 (fever*)) OR hayfever):ab,ti) AND (Epidemiology/exp OR 'epidemiological data'/exp OR epidemiology:lnk OR (prevalenc* OR inciden* OR trend* OR associat* OR comorbid* OR relat* OR correlat* OR (case NEAR/3 (control* OR comparison OR referent)) OR epidemiolog* OR cohort* OR risk* OR caus* OR (odds NEXT/1 ratio*) OR etiol* OR aetiol* OR (natural NEXT/1 histor*) OR predict* OR prognos* OR outcome* OR course*):ab,ti ) AND (child/exp OR newborn/exp OR adolescent/exp OR adolescence/exp OR 'child behavior'/de OR 'child parent relation'/de OR (adolescen* OR infan* OR newborn* OR (new NEXT/1 born*) OR baby OR babies OR neonat* OR child* OR kid OR kids OR toddler* OR teen* OR boy* OR girl* OR minors OR underag* OR (under NEXT/1 ag*) OR juvenil* OR youth* OR kindergar* OR puber* OR pubescen* OR prepubescen* OR prepubert* OR pediatric* OR paediatric* OR school* OR preschool* OR highschool*):ab,ti)

**For Medline via OvidSP**

(exp asthma/ OR (asthma* OR wheez* OR hyperresponsiv* OR hypersensit* OR (hyper ADJ (responsiv* OR sensitiv*))).ab,ti.) AND (exp Dermatitis, Atopic/ OR exp Eczema/ OR Eczem*.ab,ti. OR (atopic ADJ3 dermatit*).ab,ti.) AND (exp Rhinitis/ OR exp Conjunctivitis/ OR (rhinit* OR rhinoconjunctivit* OR conjunctivit* OR (Pollen ADJ3 Allerg*) OR Pollinos* OR hayfever* OR hay fever*).ab,ti.) AND (exp Epidemiologic Studies/ OR exp Epidemiologic Factors/ OR epidemiology.xs. OR (prevalenc* OR inciden* OR trend* OR associat* OR comorbid* OR relat* OR correlat* OR (case ADJ3 (control* OR comparison OR referent)) OR epidemiolog* OR cohort* OR risk* OR caus* OR odds ratio* OR etiol* OR aetiol* OR natural histor* OR predict* OR prognos* OR outcome* OR course*).ab,ti. ) AND (exp child/ OR exp infant/ OR (infan* OR newborn* OR new born* OR baby OR babies OR neonat* OR perinat* OR postnat* OR child* OR kid? OR toddler* OR teen* OR boy? OR girl? OR minor? OR underag* OR (under ADJ2 ag?) OR juvenil* OR youth? OR kindergar* OR puber* OR pubescen* OR prepubescen* OR prepuberty* OR pediatric* OR peadiatric* OR school* OR preschool* OR highschool* OR suckling*).ab,ti. OR ((adoles*.ab,ti. OR adolescent/) NOT exp adult/))

**PubMed publisher**

(asthma[mh] OR (asthma*[tiab] OR wheez*[tiab] OR hyperresponsiv*[tiab] OR hypersensit*[tiab] OR hyper responsiv*[tiab] OR hyper sensitiv*[tiab])) AND (Dermatitis, Atopic[mh] OR Eczema[mh] OR Eczem*[tiab] OR (atopic AND dermatit*[tiab])) AND (Rhinitis[mh] OR Conjunctivitis[mh] OR (rhinit*[tiab] OR rhinoconjunctivit*[tiab] OR conjunctivit*[tiab] OR (Pollen AND Allerg*[tiab]) OR Pollinos*[tiab] OR hayfever*[tiab] OR hay fever*[tiab])) AND (Epidemiologic Studies[mh] OR Epidemiologic Factors[mh] OR epidemiology[sh] OR (prevalenc*[tiab] OR inciden*[tiab] OR trend*[tiab] OR associat*[tiab] OR comorbid*[tiab] OR relat*[tiab] OR correlat*[tiab] OR (case AND (control*[tiab] OR comparison OR referent)) OR epidemiolog*[tiab] OR cohort*[tiab] OR risk*[tiab] OR caus*[tiab] OR odds ratio*[tiab] OR etiol*[tiab] OR aetiol*[tiab] OR natural histor*[tiab] OR predict*[tiab] OR prognos*[tiab] OR outcome*[tiab] OR course*[tiab]) ) AND (child[mh] OR infant[mh] OR (infan*[tiab] OR newborn*[tiab] OR new born*[tiab] OR baby OR babies OR neonat*[tiab] OR perinat*[tiab] OR postnat*[tiab] OR child*[tiab] OR kid* OR toddler*[tiab] OR teen*[tiab] OR boy* OR girl* OR minor* OR underag*[tiab] OR under ag* OR juvenil*[tiab] OR youth* OR kindergar*[tiab] OR puber*[tiab] OR pubescen*[tiab] OR prepubescen*[tiab] OR prepuberty*[tiab] OR pediatric*[tiab] OR peadiatric*[tiab] OR school*[tiab] OR preschool*[tiab] OR highschool*[tiab] OR suckling*[tiab]) OR ((adoles*[tiab] OR adolescent[mh]) NOT adult[mh])) AND publisher[sb]

**For Cochrane**

((asthma* OR wheez* OR hyperresponsiv* OR hypersensit* OR (hyper NEXT/1 (responsiv* OR sensitiv*))):ab,ti) AND ((eczem* OR (atopic NEAR/3 dermatit*)):ab,ti) AND ((rhinitis* OR rhinoconjunctivit* OR conjunctivit* OR (Pollen NEAR/3 Allerg*) OR Pollinos* OR ((hay) NEXT/1 (fever*)) OR hayfever):ab,ti) AND ((prevalenc* OR inciden* OR trend* OR associat* OR comorbid* OR relat* OR correlat* OR (case NEAR/3 (control* OR comparison OR referent)) OR epidemiolog* OR cohort* OR risk* OR caus* OR (odds NEXT/1 ratio*) OR etiol* OR aetiol* OR (natural NEXT/1 histor*) OR predict* OR prognos* OR outcome* OR course*):ab,ti ) AND ((adolescen* OR infan* OR newborn* OR (new NEXT/1 born*) OR baby OR babies OR neonat* OR child* OR kid OR kids OR toddler* OR teen* OR boy* OR girl* OR minors OR underag* OR (under NEXT/1 ag*) OR juvenil* OR youth* OR kindergar* OR puber* OR pubescen* OR prepubescen* OR prepubert* OR pediatric* OR paediatric* OR school* OR preschool* OR highschool*):ab,ti)

**Google scholar**

asthma eczema rhinitis prevalence|incidence|epidemiology|cohort|risk|etiology|prognosis|outcome adolescents|infants|children|newborns "family|general|primary physician|practice|doctor|care"

***Search string TWO contained the following specific terms:***

***For EMBASE:***

((asthma/exp OR wheezing/de OR (asthma* OR wheez* OR hyperresponsiv* OR hypersensit* OR (hyper NEXT/1 (responsiv* OR sensitiv*))):ab,ti) OR (eczema/de OR 'atopic dermatitis'/de OR (eczem* OR (atopic NEAR/3 dermatit*)):ab,ti) OR (rhinitis/exp OR conjunctivitis/exp OR (rhinitis* OR rhinoconjunctivit* OR conjunctivit* OR (Pollen NEAR/3 Allerg*) OR Pollinos* OR ((hay) NEXT/1 (fever*)) OR hayfever):ab,ti)) AND (Epidemiology/exp OR 'epidemiological data'/exp OR epidemiology:lnk OR (prevalenc* OR inciden* OR trend* OR associat* OR comorbid* OR relat* OR correlat* OR (case NEAR/3 (control* OR comparison OR referent)) OR epidemiolog* OR cohort* OR risk* OR caus* OR (odds NEXT/1 ratio*) OR etiol* OR aetiol* OR (natural NEXT/1 histor*) OR predict* OR prognos* OR outcome* OR course*):ab,ti ) AND (child/exp OR newborn/exp OR adolescent/exp OR adolescence/exp OR 'child behavior'/de OR 'child parent relation'/de OR (adolescen* OR infan* OR newborn* OR (new NEXT/1 born*) OR baby OR babies OR neonat* OR child* OR kid OR kids OR toddler* OR teen* OR boy* OR girl* OR minors OR underag* OR (under NEXT/1 ag*) OR juvenil* OR youth* OR kindergar* OR puber* OR pubescen* OR prepubescen* OR prepubert* OR pediatric* OR paediatric* OR school* OR preschool* OR highschool*):ab,ti) AND (Isaac OR 'Asthma and Allergies in Childhood' OR 'Asthma and Allergy in Childhood'):de,ab,ti

**For Medline via OvidSP**

((exp asthma/ OR (asthma* OR wheez* OR hyperresponsiv* OR hypersensit* OR (hyper ADJ (responsiv* OR sensitiv*))).ab,ti.) OR (exp Dermatitis, Atopic/ OR exp Eczema/ OR Eczem*.ab,ti. OR (atopic ADJ3 dermatit*).ab,ti.) OR (exp Rhinitis/ OR exp Conjunctivitis/ OR (rhinit* OR rhinoconjunctivit* OR conjunctivit* OR (Pollen ADJ3 Allerg*) OR Pollinos* OR hayfever* OR hay fever*).ab,ti.)) AND (exp Epidemiologic Studies/ OR exp Epidemiologic Factors/ OR epidemiology.xs. OR (prevalenc* OR inciden* OR trend* OR associat* OR comorbid* OR relat* OR correlat* OR (case ADJ3 (control* OR comparison OR referent)) OR epidemiolog* OR cohort* OR risk* OR caus* OR odds ratio* OR etiol* OR aetiol* OR natural histor* OR predict* OR prognos* OR outcome* OR course*).ab,ti. ) AND (exp child/ OR exp infant/ OR (infan* OR newborn* OR new born* OR baby OR babies OR neonat* OR perinat* OR postnat* OR child* OR kid? OR toddler* OR teen* OR boy? OR girl? OR minor? OR underag* OR (under ADJ2 ag?) OR juvenil* OR youth? OR kindergar* OR puber* OR pubescen* OR prepubescen* OR prepuberty* OR pediatric* OR peadiatric* OR school* OR preschool* OR highschool* OR suckling*).ab,ti. OR ((adoles*.ab,ti. OR adolescent/) NOT exp adult/)) AND (Isaac OR "Asthma and Allergies in Childhood" OR "Asthma and Allergy in Childhood").ab,ti.

**Pubmed publisher**

((asthma[mh] OR (asthma*[tiab] OR wheez*[tiab] OR hyperresponsiv*[tiab] OR hypersensit*[tiab] OR (hyper responsiv*[tiab] OR hypersensitiv*[tiab])) OR (Dermatitis, Atopic[mh] OR Eczema[mh] OR Eczem*[tiab] OR (atopic AND dermatit*[tiab])) OR (Rhinitis[mh] OR Conjunctivitis[mh] OR (rhinit*[tiab] OR rhinoconjunctivit*[tiab] OR conjunctivit*[tiab] OR (Pollen AND Allerg*[tiab]) OR Pollinos*[tiab] OR hayfever*[tiab] OR hay fever*[tiab]))) AND (Epidemiologic Studies[mh] OR Epidemiologic Factors[mh] OR epidemiology[sh] OR (prevalenc*[tiab] OR inciden*[tiab] OR trend*[tiab] OR associat*[tiab] OR comorbid*[tiab] OR relat*[tiab] OR correlat*[tiab] OR (case AND (control*[tiab] OR comparison OR referent)) OR epidemiolog*[tiab] OR cohort*[tiab] OR risk*[tiab] OR caus*[tiab] OR odds ratio*[tiab] OR etiol*[tiab] OR aetiol*[tiab] OR natural histor*[tiab] OR predict*[tiab] OR prognos*[tiab] OR outcome*[tiab] OR course*[tiab]) ) AND (child[mh] OR infant[mh] OR (infan*[tiab] OR newborn*[tiab] OR new born*[tiab] OR baby OR babies OR neonat*[tiab] OR perinat*[tiab] OR postnat*[tiab] OR child*[tiab] OR kid* OR toddler*[tiab] OR teen*[tiab] OR boy* OR girl* OR minor* OR underag*[tiab] OR (under ag*) OR juvenil*[tiab] OR youth* OR kindergar*[tiab] OR puber*[tiab] OR pubescen*[tiab] OR prepubescen*[tiab] OR prepuberty*[tiab] OR pediatric*[tiab] OR peadiatric*[tiab] OR school*[tiab] OR preschool*[tiab] OR highschool*[tiab] OR suckling*[tiab]) OR ((adoles*[tiab] OR adolescent[mh]) NOT adult[mh])) AND (Isaac OR "Asthma and Allergies in Childhood" OR "Asthma and Allergy in Childhood") AND publisher[sb]

**For Cochrane**

(((asthma* OR wheez* OR hyperresponsiv* OR hypersensit* OR (hyper NEXT/1 (responsiv* OR sensitiv*))):ab,ti) OR ((eczem* OR (atopic NEAR/3 dermatit*)):ab,ti) OR ((rhinitis* OR rhinoconjunctivit* OR conjunctivit* OR (Pollen NEAR/3 Allerg*) OR Pollinos* OR ((hay) NEXT/1 (fever*)) OR hayfever):ab,ti)) AND ((prevalenc* OR inciden* OR trend* OR associat* OR comorbid* OR relat* OR correlat* OR (case NEAR/3 (control* OR comparison OR referent)) OR epidemiolog* OR cohort* OR risk* OR caus* OR (odds NEXT/1 ratio*) OR etiol* OR aetiol* OR (natural NEXT/1 histor*) OR predict* OR prognos* OR outcome* OR course*):ab,ti ) AND ((adolescen* OR infan* OR newborn* OR (new NEXT/1 born*) OR baby OR babies OR neonat* OR child* OR kid OR kids OR toddler* OR teen* OR boy* OR girl* OR minors OR underag* OR (under NEXT/1 ag*) OR juvenil* OR youth* OR kindergar* OR puber* OR pubescen* OR prepubescen* OR prepubert* OR pediatric* OR paediatric* OR school* OR preschool* OR highschool*):ab,ti) AND (Isaac OR 'Asthma and Allergies in Childhood' OR 'Asthma and Allergy in Childhood'):ab,ti

**Google scholar**

asthma|wheezing|hyperresponsive|hypersensitivity|eczema|"atopic dermatitis"|rhinitis|conjunctivitis|"Pollen Allergy|allergies"|Pollinos|"hay fever"|hayfever prevalence|incidence|comorbidity|comorbidities|epidemiology|epidemiological infants|children Isaac
